# Eikonal equations and pathwise solutions to fully non-linear SPDEs

**DOI:** 10.1007/s40072-016-0087-9

**Published:** 2016-12-03

**Authors:** Peter K. Friz, Paul Gassiat, Pierre-Louis Lions, Panagiotis E. Souganidis

**Affiliations:** 1 Institut für Mathematik, Technische Universität Berlin, Straße des 17. Juni 136, 10623 Berlin, Germany; 2 Weierstraß-Institut für Angewandte Analysis und Stochastik, Mohrenstraße 39, 10117 Berlin, Germany; 3 CEREMADE, Université de Paris-Dauphine, Place du Maréchal-de-Lattre-de-Tassigny, 75775 Paris Cedex 16, France; 4 Collège de France and CEREMADE, Université de Paris-Dauphine, 1, Place Marcellin Berthelot, 75005 Paris Cedex 5, France; 5 Department of Mathematics, University of Chicago, Chicago, IL 60637 USA

**Keywords:** Fully non-linear stochastic partial differential equations, Eikonal equations, Pathwise stability, Rough paths, 35R99, 60H15

## Abstract

We study the existence and uniqueness of the stochastic viscosity solutions of fully nonlinear, possibly degenerate, second order stochastic pde with quadratic Hamiltonians associated to a Riemannian geometry. The results are new and extend the class of equations studied so far by the last two authors.

## Introduction

The theory of stochastic viscosity solutions, including existence, uniqueness and stability, developed by two of the authors (Lions and Souganidis [4–9]) is concerned with pathwise solutions to fully nonlinear, possibly degenerate, second order stochastic pde, which, in full generality, have the form

1 $$\begin{aligned} {\left\{ \begin{array}{ll} du=F(D^{2}u,Du, u, x, t) dt + \sum _{i=1}^{d}H_{i}\left( Du, u, x\right) d\xi ^{i} \ \text { in } \ \mathbb {R}^N \times (0,T],\\ u=u_0 \ \text {on} \ \mathbb {R}^N \times \{0\}; \end{array}\right. } \end{aligned}$$

here *F* is degenerate elliptic and $\xi =(\xi ^1,\ldots , \xi ^d)$ is a continuous path. A particular example is a *d*-dimensional Brownian motion, in which case (1) should be interpreted in the Stratonovich sense. Typically, $u \in \mathrm {BUC}(\mathbb {R}^N \times [0,T])$, the space of bounded uniformly continuous real-valued functions on $\mathbb {R}^N \times [0,T]$.

For the convenience of the reader we present a quick general overview of the theory: The Lions–Souganidis theory applies to rather general paths when $H=H(p)$ and, as established in [6, 9], there is a very precise trade off between the regularity of the paths and *H*. When $H=H(p,x)$ and $d=1$, the results of [9] deal with general continuous, including Brownian paths, and the theory requires certain global structural conditions on *H* involving higher order (up to three) derivatives in *x* and *p*. Under similar conditions, Lions and Souganidis [10] have also established the wellposedness of (1) for $d>1$ and Brownian paths. For completeness we note that, when $\xi$ is smooth, for example $\text {C}^1$, (1) falls within the scope of the classical Crandall–Lions viscosity theory—see, for example, Crandall et al. [2].

The aforementioned conditions are used to control the length of the interval of existence of smooth solutions of the so-called doubled equation

2 $$\begin{aligned} dw=(H(D_x w, x)-H(-D_y w,y))d\xi \ \text { in } \ \mathbb {R}^N \times (t_0 - h_*, t_0+h_*) \end{aligned}$$

with initial datum

3 $$\begin{aligned} w(x,y,t_0)=\lambda |x-y|^2 \end{aligned}$$

as $\lambda \rightarrow \infty$ and uniformly for $|x-y|$ appropriately bounded.

It was, however, conjectured in [9] that, given a Hamiltonian *H*, it may be possible to find initial data other than $\lambda |x-y|^2$ for the doubled equation, which are better adapted to *H*, thus avoiding some of the growth conditions. As a matter of fact this was illustrated by an example when $N=1$.

In this note we follow up on the remark above about the structural conditions on *H* and identify a better suited initial data for (2) for the special class of quadratic Hamiltonians of the form

4 $$\begin{aligned} H(p,x):= (g^{-1}(x)p,p)= \sum _{i,j=1}^{N}g^{i,j}\left( x\right) p_{i}p_{j}, \end{aligned}$$

which are associated to a Riemannian geometry in $\mathbb {R}^N$ and do not satisfy the conditions mentioned earlier, where

5 $$\begin{aligned} g=( g_{i,j})_{1\le i,j\le N} \in C^2(\mathbb {R}^N; {\mathcal {S}}^N) \end{aligned}$$

is positive definite, that is there exists $C>0$ such that, for all $w\in \mathbb {R}^N$,

6 $$\begin{aligned} \frac{1}{C} \left| w\right| ^{2} \le \sum _{i,j} g_{i,j}\left( x\right) w^{i}w^{j} \le C \left| w\right| ^{2}. \end{aligned}$$

It follows from (4) and (6) that *g* is invertible and $g^{-1}=( g^{i,j})_{1\le i,j\le N}\in C^2(\mathbb {R}^N; {\mathcal {S}}^N)$ is also positive definite; here ${\mathcal {S}}^N$ is the space of $N\times N$-symmetric matrices and (*p*, *q*) denotes the usual inner product of the vectors *p*, *q* $\in \mathbb {R}^N$. When dealing with (1) it is necessary to strengthen (5) and we assume that

7 $$\begin{aligned} g, g^{-1} \in C^2_b(\mathbb {R}^N; {\mathcal {S}}^N), \end{aligned}$$

where $C^2_b(\mathbb {R}^N; {\mathcal {S}}^N)$ is the set of functions bounded in $C^2(\mathbb {R}^N; {\mathcal {S}}^N)$. Note that in this case (6) is implied trivially.

The distance $d_{g}\left( x,y\right)$ with respect to *g* of two points $x,y \in \mathbb {R}^N$ is given by

$$\begin{aligned} d_{g}\left( x,y\right) := \inf \left\{ \int _{0}^{1} \frac{1}{2} (g(\gamma _t) \dot{\gamma }_t, \dot{\gamma }_t)^{1/2} dt : \gamma \in C^1 ([0,1],{\mathbb {R}}^{N}), \gamma _0 = x, \gamma _1 = y \right\} , \end{aligned}$$

and their associated “energy” is

8 $$\begin{aligned} e_{g}( x,y) := d_{g}^2( x,y)=\inf \left\{ \int _{0}^{1} \frac{1}{4} (g(\gamma _t) \dot{\gamma }_t, \dot{\gamma }_t) dt : \gamma \in C^1 ([0,1],{\mathbb {R}}^{N}), \gamma _0 = x, \gamma _1 = y \right\} . \end{aligned}$$

Note that, if $g=I$ the identity $N\times N$ matrix in $\mathbb {R}^N$, then $d_I(x,y)= \tfrac{1}{2}|x-y|$, the usual Euclidean distance, and $e_{I}\left( x,y\right) = \tfrac{1}{4} |x-y|^2;$ more generally, (6) implies, with $C=c^2$ and for all $x, y\in {{\mathbb {R}}}^{N}$,

$$\begin{aligned} \frac{1}{2c} |x-y| \le d_{g}\left( x,y\right) \le \frac{1}{2} c |x-y|. \end{aligned}$$

In addition, we assume that

9 $$\begin{aligned} \text { there exists }\Upsilon > 0 \text { such that }e_g \in C^1\left( \left\{ (x,y) \in {\mathbb {R}}^{N} \times \mathbb {R}^N: d_g(x,y) < \Upsilon \right\} \right) ; \end{aligned}$$

in the language of differential geometry (9) is the same as to say that the manifold $( {\mathbb {R}} ^N, g)$ has strictly positive injectivity radius. We remark that (7) is sufficient for (9) (see, for example, Proposition 4.3), though (far) from necessary.

We continue with some terminology and notation that we will need in the paper. We write $I_N$ for the identity matrix in $\mathbb {R}^N$. A modulus is a nondecreasing, subadditive function $\omega :[0,\infty ) \rightarrow [0,\infty )$ such that $\lim _{r\rightarrow 0}\omega \left( r\right) = \omega (0) = 0$. We write $u\in \mathrm {UC_{g}}\left( {\mathbb {R}}^{N}\right)$ if $\left| u\left( x\right) -u\left( y\right) \right| \le \omega \left( d_{g}\left( x,y\right) \right)$ for some modulus $\omega$, and, given $u\in \mathrm {UC_{g}}\left( {\mathbb {R}}^{N}\right)$, we denote by $\omega _{u}$ its modulus. When *u* is also bounded, we write $u \in \mathrm {BUC_g}\left( {\mathbb {R}}^{N}\right)$ and may take its modulus bounded. We denote by $\mathrm {USC}$ (resp. $\mathrm {LSC}$) the set of upper- (resp. lower) semicontinuous functions in $\mathbb {R}^N$, and $\mathrm {BUSC}$ (resp. $\mathrm {BLSC}$) is the set of bounded functions in $\mathrm {USC}$ (resp. $\mathrm {LSC}$). For a bounded continuous function $u:\mathbb {R}^k\rightarrow \mathbb {R}$, for some $k\in \mathbb {N}$, and $A\subset \mathbb {R}^k$, $\Vert u\Vert _{\infty , A}:=\sup _A |u|$. If $a,b\in \mathbb {R}$, then $a\wedge b:=\min (a,b)$, $a_+:=\max (a,0)$ and $a_-:=\max (-a,0)$. Given a modulus $\omega$ and $\lambda >0$, we use the function $\theta : (0,\infty ) \rightarrow (0,\infty )$ defined by

10 $$\begin{aligned} \theta (\omega ,\lambda ):=\sup _{r\ge 0}\{\omega (r)-\lambda r^{2}/2\}; \end{aligned}$$

and observe that, in view of the assumed properties of the modulus,

11 $$\begin{aligned} \lim _{\lambda \rightarrow \infty } \theta (\omega ;\lambda ) = 0. \end{aligned}$$

Finally, for $k \in \mathbb {N}$, $C^k_0 ([0,T];{\mathbb {R}}):=\{ \zeta \in C^k([0,T];{\mathbb {R}}): \zeta _0=0 \}$ and, of for any two $\zeta , \xi \in C_0 ([0,T];{\mathbb {R}})$, we set

12 $$\begin{aligned} \Delta _{T}^{+}:=\max _{s\le T}(\xi _{s}-\zeta _{s})\ge 0 \ \text { and } \ \Delta _{T}^{-}:=\max _{s\le T}\{-(\xi _{s}-\zeta _{s})\}\ge 0. \end{aligned}$$

We review next the approach taken in [4–9] to define solutions to (1). The key idea is to show that the solutions of the initial value problems with smooth paths, which approximate locally uniformly the given continuous one, form a Cauchy family in $BUC(\mathbb {R}^N\times [0,T])$ for all $T>0$, and thus converge to a limit which is independent of the regularization. This limit is considered as the solution to (1). It follows that the solution operator for (1) is the extension in the class of continuous paths of the solution operator for smooth paths. Then [4–9] introduced an intrinsic definition for a solution, called stochastic viscosity solution, which is satisfied by the uniform limit. Moreover, it was shown that the stochastic viscosity solutions satisfy a comparison principle and, hence, are intrinsically unique and can be constructed by the classical Perron’s method (see [9, 13] for the complete argument). The assumptions on the Hamiltonians mentioned above were used in these references to obtain both the Cauchy property and the intrinsic uniqueness.

To prove the Cauchy property the aforementioned references consider the solutions to (1) corresponding to two different smooth paths $\zeta _1$ and $\zeta _2$ and establish an upper bound for the $\sup$-norm of their difference. The classical viscosity theory provides immediately such a bound, which, however, depends on the $L^1$-norm of $\dot{\zeta }_1 -\dot{\zeta }_2$. Such a bound is, of course, not useful since it blows up, as the paths approximate the given continuous path $\xi$. The novelty of the Lions-Souganidis theory is that it is possible to obtain far better control of the difference of the solutions based on the $\sup$-norm of $\zeta _1 -\zeta _2$ at the expense of some structural assumptions on *H*. In the special case of (1) with $F=0$ and *H* independent of *x*, a sharp estimate was obtained in [9]. It was also remarked there that such bound cannot be expected to hold for spatially dependent Hamiltonians without additional restrictions.

In this note we take advantage of the very particular quadratic structure of *H* and obtain a local in time bound on the difference of two solutions with smooth paths. That the bound is local is due to the need to deal with smooth solutions of the Hamilton-Jacobi part of the equation. Quadratic Hamiltonians do not satisfy the assumptions in [9]. Hence, the results here extend the class of (1) for which there exists a well posed solution. The bound obtained is also used to give an estimate for the solutions to (1), (4) corresponding to different merely continuous paths as well as a modulus of continuity.

Next we present the results and begin with the comparison of solutions with smooth and different paths. Since the assumptions on the metric *g* are slightly stronger in the presence of the second order term in (1), we state two theorems. The first is for the first-order problem

13 $$\begin{aligned} {\left\{ \begin{array}{ll} du - (g^{-1}(x) Du, Du)d\xi =0 \ \text{ in } \ \mathbb {R}^N \times (0, T],\\ u=u_0 \ \text {on} \ \mathbb {R}^N \times \{0\}, \end{array}\right. } \end{aligned}$$

and the second for (1) with *H* given by (4). Then we discuss the extension property and the comparison for general paths.

We first assume that we have smooth driving signals and estimate the difference of solutions. Since we are working with “classical” viscosity solutions, we write $u_t$ and $\dot{\xi }_t$ in place of of *du* and $d\xi _t$.

### Theorem 1.1

Assume (5), (6) and (9) and let $\xi ,\zeta \in C_0^{1}([0,T];{\mathbb {R}})$ and $u_{0},v_{0}\in \mathrm {BUC_g(\mathbb {R}^N)}$. Let $u \in \mathrm {BUSC}(\mathbb {R}^N \times [0,T])$ and $v \in \mathrm {BLSC}(\mathbb {R}^N \times [0,T])$ be respectively viscosity sub- and super-solutions to

$$\begin{aligned} u_t-(g^{-1}(x)Du, Du) \dot{\xi }\le 0 \ \mathrm { in } \ \mathbb {R}^N \times (0,T] \ \ \ u(\cdot ,0 )\le u_{0} \ \mathrm { on } \ \mathbb {R}^N, \end{aligned}$$

and

$$\begin{aligned} v_t-(g^{-1}(x)Dv, Dv)\dot{\zeta }\ge 0 \ \mathrm { in } \ \mathbb {R}^N \times (0,T] \ \ \ v(\cdot , 0 )\ge v_{0} \ \mathrm { on } \ \mathbb {R}^N \end{aligned}$$

Then, if

14 $$\begin{aligned}&\Delta _{T}^{+}+\Delta _{T}^{-}\,<\frac{1}{2\left( \left\| u_0 \right\| _{\infty ; {\mathbb {R}}^{N}}+\left\| v_0 \right\| _{\infty ; { \mathbb {R}}^{N}}\right) } \Upsilon ^{2}, \end{aligned}$$

15 $$\begin{aligned}&\sup _{ \mathbb {R}^N \times [0,T]} \left( u-v\right) \;\le \;\; \sup _{\mathbb {R}^N}\left( u_{0}-v_{0}\right) +\theta \left( \omega _{u_{0}} \wedge \omega _{v_{0}},\frac{1 }{\Delta _{T}^{+}}\right) . \end{aligned}$$

We consider now the second-order fully nonlinear equation (1) with quadratic Hamiltonians, that is the initial value problem

16 $$\begin{aligned} \left\{ \begin{array}{ll} du=F\left( D^{2}u,Du,u,x,t\right) dt +(g^{-1}(x) Du, Du)d\xi &{} \hbox { in } {\mathbb {R}}^{N} \times 0,T],\\ u(\cdot ,0 )=u_{0}\in \mathrm {BUC}( {\mathbb {R}}^{N}), &{} \end{array} \right. \end{aligned}$$

and introduce assumptions on *F* in order to have a result similar to Theorem 1.1.

In order to be able to have some checkable structural conditions on *F*, we find it necessary to replace (5) and (9) by the stronger conditions (7) and

17 $$\begin{aligned} \text {there exists } \Upsilon >0 \text { such that } D^2d_g^2 \ \text {is bounded on } \{(x,y): d_g(x,y) <\Upsilon \}. \end{aligned}$$

As far as $F\in \text { C} ( {\mathcal {S}}^N \times {\mathbb {R}} ^N \times { \mathbb {R}} ^N\times [0,T]; {\mathbb {R}} )$ is concerned we assume that it is degenerate elliptic, that is for all $X, Y \in \mathcal {S}^N$ and $(p,r,x,t) \in {\mathbb {R}} ^N \times \mathbb {R}^N\times [0,T]$,

18 $$\begin{aligned} F(X,p,r,x,t)\le F(Y,p,r,x,t) \text { if } X\le Y, \end{aligned}$$

Lipschitz continuous in *r*, that is

19 $$\begin{aligned} \text { there exists }L>0 \text { such that } |F(X,p,r,x,t)-F(X,p,s,x,t)| \le L|s-r|, \end{aligned}$$

bounded in (*x*, *t*), in the sense that

20 $$\begin{aligned} \sup _{{\mathbb {R}} ^N\times [0,T]} |F(0,0,0,\cdot , \cdot )| < \infty , \end{aligned}$$

and uniformly continuous for bounded (*X*, *p*, *r*), that is, for any $R>0$,

21 $$\begin{aligned} F \text { is uniformly continuous on } M_{R} \times B_{R} \times [-R,R] \times {\mathbb {R}} ^N \times [0,T], \end{aligned}$$

where $M_{R}$ and $B_{R}$ are respectively the balls of radius *R* in ${\mathcal {S}}^N$ and ${\mathbb {R}}^{N}$.

Similarly to the classical theory of viscosity solutions, it is also necessary to assume something more about the joint continuity of *F* in *X*, *p*, *x*, namely that

22 $$\begin{aligned} {\left\{ \begin{array}{ll} \text { for each }R>0 \text { there exists a modulus } \omega _{F,R}\text { such that, for all }\alpha , \varepsilon >0\text { and uniformly on}\\ t\in [0,T] \text { and } r\in [-R,R], \\ F(X, \alpha D_{x}d_g^{2}(x,y), r,x,t)-F(Y,-\alpha D_{y}d_g^{2}(x,y),r,y,t)\le \omega _{F,R}(\alpha d_g^{2}(x,y)+d_g(x,y) + \varepsilon ), \\ \text { whenever } d_g(x,y) < \Upsilon \text { and } X,Y\in {\mathcal {S}}^N \text { are such that, for } A=D_{(x,y)}^{2}d_g^2(x,y), \\ -(\alpha ^2\varepsilon ^{-1} + \Vert A\Vert ) \left( \begin{array}{cc} I\; &{} 0 \\ 0\; &{} I \end{array} \right) \le \left( \begin{array}{cc} X &{} 0 \\ 0 &{} -Y \end{array} \right) \le \alpha A+\varepsilon A^{2}.\\ \end{array}\right. } \end{aligned}$$

Note that in the deterministic theory the above assumption is stated using the Euclidean distance. Here it is convenient to use $d_g$ and as a result we find it necessary to strengthen the assumptions on the metric *g*.

To simplify the arguments below, instead of (19), we will assume that *F* monotone in *r*, that is

23 $$\begin{aligned} \text { there exists }\rho >0\text { such that } F(X,p,r,x,t)-F(X,p,s,x,t) \ge \rho (s-r) \text { whenever } s\ge r; \end{aligned}$$

this is, of course, not a restriction since we can always consider the change $u(x,t)=e^{(L+\rho )t}v(x,t)$, which yields an equation for *v* with a new *F* satisfying (23) and path $\xi '$ such that $\dot{\xi }'_t= e^{(L+\rho )t}\dot{\xi }_t$.

To state the result we introduce some additional notation. For $\gamma >0$, we write

24 $$\begin{aligned} \tilde{\theta }(\omega ;\gamma ):=\sup _{r\ge 0}(\omega (r)- \frac{\gamma }{2}r), \end{aligned}$$

and, for $$\xi , \zeta \in C^1([0,\infty );\mathbb {R})$$,

25 $$\begin{aligned} \Delta _{T}^{\gamma ,+}:=\sup _{t\le T}\int _{0}^{t}e^{\gamma s}(\dot{\xi } _{s}-\dot{\zeta }_{s})ds \text { and } \Delta _{T}^{\gamma ,-}:=\sup _{t\le T}\left[ -\int _{0}^{t}e^{\gamma s}(\dot{\xi }_{s}-\dot{\zeta }_{s})ds\right] . \end{aligned}$$

Finally, for bounded $u_0, v_0:\mathbb {R}^N\rightarrow \mathbb {R}$, let

$$\begin{aligned} K:=\frac{2}{\rho }\left[ \sup _{(x,t) \in \mathbb {R}^N \times [0,T]}F(0,0,0,x,t)+\Vert u_{0}\Vert _{\infty ;{\mathbb { R}}^{N}}+\Vert v_{0}\Vert _{\infty ;{\mathbb {R}}^{N}}\right] . \end{aligned}$$

We have:

### Theorem 1.2

Assume (7), (17)–(22), and let $\xi ,\zeta \in C_0^{1}([0,T];{\mathbb {R}})$ $u_{0},v_{0}\in \mathrm {BUC}({\mathbb {R}}^{N})$ and $T>0$. If *u* $\in \mathrm {BUSC}_g( \mathbb {R}^N \times [0,T])$ and $v\in$ $\mathrm {BLSC}( \mathbb {R}^N \times [0,T])$ are respectively viscosity sub- and super-solutions of

26 $$\begin{aligned} u_t-F(D^{2}u,Du,u,x,t)-(g^{-1}(x) Du, Du)\dot{\xi }\le 0\ \hbox { in }{ \mathbb {R}}^{N}\times (0,T]\qquad u(0,\cdot )\le u_{0}\ \text { on}\ { \mathbb {R}}^{N}, \end{aligned}$$

and

27 $$\begin{aligned} v_t-F(D^{2}v,Dv,v,x,t)-(g^{-1}(x) Dv, Dv)\dot{\zeta }\ge 0\ \text{ in } { \mathbb {R}}^{N}\times (0,T]\qquad v(0,\cdot )\ge u_{0}\ \text { on}\ { \mathbb {R}}^{N}, \end{aligned}$$

then, if $$\Gamma := \{ \gamma >0: \Delta _{T}^{\gamma ,+}+\Delta _{T}^{\gamma ,-}<\frac{\Upsilon ^{2}}{4K} \ \text {and} \ \Delta _{T}^{\gamma ,-} <1\ \},$$

28 $$\begin{aligned} {\left\{ \begin{array}{ll} \sup _{ \mathbb {R}^N \times [0,T]} \left( u-v\right) \le \sup _{\mathbb {R}^N} \left( u_{0}-v_{0}\right) _{+} \\ +\inf _{\gamma \in \Gamma }\left[ \theta \left( \omega _{u_{0}}\wedge \omega _{v_{0}},\frac{1}{\Delta _{T}^{\gamma ,+}}\right) +\frac{1}{\rho } \tilde{\theta }( \omega _{F,K};\gamma ) + \frac{1}{\rho } \omega _{F,K}( 2 (K( \Delta _{T}^{\gamma ,+}+\Delta _{T}^{\gamma ;-}) )^{1/2} \right] . \end{array}\right. } \end{aligned}$$

Under their respective assumptions, Theorems 1.1 and 1.2 imply that, for paths $\xi \in C^1([0,\infty );\mathbb {R})$ and $g\in \text {BUC}_g(\mathbb {R}^N)$, the initial value problems (13) and (16) have well-defined solution operators

$$\begin{aligned} \mathcal {S} :(u_{0},\xi )\mapsto u\equiv \mathcal {S}^{\xi }\left[ u_{0}\right] . \end{aligned}$$

The main interest in the estimates (15) and (28) is that they provide a unique continuous extension of this solution operator to all $\xi \in C([0,\infty );\mathbb {R})$. Since the proof is a simple reformulation of (15) and (28), we omit it.

### Theorem 1.3

Under the assumptions of Theorems 1.1 and 1.2, the solution operator $\mathcal {S}:BUC(\mathbb {R}^N) \times C^1([0,\infty );\mathbb {R}) \rightarrow BUC(\mathbb {R}^N \times [0,T])$ admits a unique continuous extension to $\bar{\mathcal {S}}:BUC(\mathbb {R}^N) \times C([0,\infty );\mathbb {R}) \rightarrow BUC(\mathbb {R}^N \times [0,T]).$ In addition, for each $T>0$, there exists a nondecreasing $\Phi : [0,\infty ) \rightarrow [0,\infty ]$, depending only on *T* and the moduli and $\sup$-norms of $u_0, v_0 \in \mathrm {BUC_g}$, such that $\lim _{r\rightarrow 0}\Phi \left( r\right) = \Phi (0) = 0$, and, for all $\xi ,\zeta \in C([0,T];{\mathbb {R}})$,

29 $$\begin{aligned} \left\| \mathcal {S}^{\xi }\left[ u_{0}\right] -\mathcal {S}^{\zeta }\left[ v_{0}\right] \right\| _{\infty ; {\mathbb {R}}^{N} \times [0,T]}\le \Vert u_{0}-v_{0}\Vert _{\infty ;{\mathbb {R}}^{N}}+\Phi \left( \left\| \xi -\zeta \right\| _{\infty ;\left[ 0,T\right] }\right) . \end{aligned}$$

We also remark that for both problems the proofs yield a, uniform in $t \in [0,T]$ and $\Vert \xi -\zeta \Vert _{\infty ;[0,T]}$, estimate for $u(x,t) - v(y,t)$. Applied to the solutions of (13) and (16), this yields a (spatial) modulus of continuity which depends only on the initial datum, *g* and *F* but not $\xi$. This allows to see (as in [3–9]) that $\mathcal {S}$ and then $\bar{\mathcal {S}}$ indeed takes values in $BUC(\mathbb {R}^N \times [0,T])$.

An example of *F* that satisfies the assumptions of Theorem 1.2 is the Hamilton–Jacobi–Isaacs operator

30 $$\begin{aligned} F\left( M,p,r,x,t\right) = \inf _\alpha \sup _\beta \left\{ \mathrm {tr}\left( \sigma _{\alpha \beta } \sigma _{\alpha \beta } ^{T}\left( p,x\right) M\right) +b_{\alpha \beta }\left( p,x\right) - c_{\alpha \beta } (x) r \right\} , \end{aligned}$$

with

31 $$\begin{aligned} \sigma , b, c \text { bounded uniformly in }\alpha , \beta \end{aligned}$$

such that, for some modulus $\omega$ and constant $C>0$ and uniformly in $\alpha , \beta$,

32 $$\begin{aligned} \left| \sigma _{\alpha \beta } (p,x)-\sigma _{\alpha \beta } (q,y)\right| \le C(\left| x-y\right| +\frac{\left| p-q\right| }{\left| p\right| +\left| q\right| }), \end{aligned}$$

and

33 $$\begin{aligned}&\left| b_{\alpha \beta }(p,x)-b_{\alpha \beta }(q,y)\right| \le \omega ((1+ |p| + |q|)|x-y|+ |p-q|),\nonumber \\&\quad \left| c_{\alpha \beta }(x) - c_{\alpha \beta }(y) \right| \le \omega (|x-y|). \end{aligned}$$

The paper is organized as follows. In the next section we prove Theorem 1.1. Section 3 is about the proof of Theorem 1.2. In the last section we state and prove a result showing that (7) implies (17) and verify that (30) satisfies the assumptions of Theorem 1.2.

## The first order case: the proof of Theorem 1.1

We begin by recalling without proof the basic properties of the Riemannian energy $e_g$ which we need in this paper. For more discussion we refer to, for example, [12] and the references therein.

### Proposition 2.1

Assume (5), (6) and (9). The Riemannian energy $e_g$ defined by (8) is (locally) absolutely continuous, almost everywhere differentiable and satisfies the Eikonal equations

34 $$\begin{aligned} (g^{-1}(y)D_{y}e_g, D_{y}e_g)=(g^{-1}(x) D_{x}e_g, D_{x}e_g)=e_g\left( x,y\right) , \end{aligned}$$

on a subset *E* of $\mathbb {R}^N \times \mathbb {R}^N$ of full measure. Moreover, $\left\{ (x,y) \in \mathbb {R}^N\times \mathbb {R}^N : d_g(x,y) < \Upsilon \right\} \subset E.$

The next lemma, which is based on (34) and the properties of *g*, is about an observation which plays a vital role in the proofs.

To this end, for $x,y \in {\mathbb {R}} ^N$, $\lambda >0$ and $\xi ,\zeta \in \text {C}_0^1([0,T])$, we set

35 $$\begin{aligned} \Phi ^{\lambda } (x,y,t):= \frac{\lambda e_g(x,y)}{1-\lambda (\xi _{t}-\zeta _{t})}. \end{aligned}$$

### Lemma 2.2

Assume (5), (6) and (9) and choose $\lambda <1/\Delta ^+ _{T}$. Then

36 $$\begin{aligned} \frac{\lambda e_g}{1+\lambda \Delta ^-_{T}} \le \Phi ^\lambda \le \frac{\lambda e_g}{1-\lambda \Delta ^+_{T}} \ \text { on } \ \mathbb {R}^N \times \mathbb {R}^N \times [0,T]. \end{aligned}$$

In addition, in the set $\{ (x,y)\in {\mathbb {R}} ^N \times {\mathbb {R}} ^N: d_g(x,y) < {\Upsilon } \}$, $\Phi ^{\lambda }$ is a classical solution of

37 $$\begin{aligned} w_t =(g^{-1}(x)D_x w, D_x w) \dot{\xi }- (g^{-1}(y)D_y w, D_y w)\dot{\zeta }. \end{aligned}$$

### Proof

The first inequality is immediate from the definition (25) of $\Delta _T^{ \pm }$. To prove (37), we observe that, in view of Proposition 2.1, we have

$$\begin{aligned} \Phi _t^{\lambda }&=\frac{\lambda ^{2}e_g(x,y)}{(1-\lambda (\xi _{t}-\zeta _{t}))^{2}}(\dot{\xi }_{t}-\dot{\zeta }_{t}), \\ \end{aligned}$$

and

$$\begin{aligned} (g^{-1}(x)D_x \Phi , D_x \Phi )&=\frac{ \lambda ^{2} }{(1-\lambda (\xi _{t}-\zeta _{t}))^{2}} (g^{-1}(x)D_x e_g, D_x e_g)\\&=\frac{ \lambda ^{2}e_g(x,y)}{(1-\lambda (\xi _{t}-\zeta _{t}))^{2}} \\&=\frac{ \lambda ^{2} }{(1-\lambda (\xi _{t}-\zeta _{t}))^{2}} (g^{-1}(y)D_y e_g, D_y e_g) \\&= (g^{-1}(y)D_y \Phi , D_y \Phi ) \end{aligned}$$

Hence, whenever $d_g( x,y)<\Upsilon$, the claim follows. $\square$

The proof of Theorem 1.1 follows the standard procedure of doubling variables. The key idea introduced in [5] is to use special solutions of the Hamiltonian part of the equation as test functions in all the comparison type-arguments, instead of the typical $\lambda |x-y|^2$ used in the “deterministic” viscosity theory. As already pointed out earlier, in the case of general Hamiltonians, the construction of the test functions in [5] is tedious and requires structural conditions on *H*. The special form of the problem at hand, however, yields easily such tests functions, which are provided by Lemma 2.2.

### Proof of Theorem 1.1

To prove (15) it suffices to show that, for all $\lambda$ in a left-neighborhood of $(\Delta ^+ _{T})^{-1}$, that is for $\lambda \in ((\Delta ^+ _{T})^{-1}-\epsilon ,(\Delta ^+ _{T})^{-1})$ for some $\epsilon >0$, and $x,y \in \mathbb {R}^N$ and $t\in [0,T]$,

38 $$\begin{aligned} u(x,t)-v(y,t)&\le \Phi ^{\lambda }(x,y,t)+\sup _{x^{\prime },y^{\prime } \in \mathbb {R}^N}\left( u_{0}(x^{\prime })-v_{0}(y^{\prime })-\lambda e_g(x^{\prime },y^{\prime })\right) \nonumber \\&\le \Phi ^{\lambda }(x,y,t)+\sup _{\mathbb {R}^N} \left( u_0-v_0\right) \nonumber \\&\quad +\sup _{x^{\prime },y^{\prime }\in \mathbb {R}^N}\left( v_{0}(x^{\prime })-v_{0}(y^{\prime })-\lambda e_g(x^{\prime },y^{\prime })\right) . \end{aligned}$$

Indeed taking $x=y$ in (38) we find

$$\begin{aligned} u(x,t)-v(x,t)\le & {} \sup _{\mathbb {R}^N} \left( u_0-v_0\right) +\sup _{x^{\prime },y^{\prime } \in \mathbb {R}^N}\left( v_{0}(x^{\prime })-v_{0}(y^{\prime })-\lambda d^{2}(x^{\prime },y^{\prime })/2\right) _{+} \\\le & {} \sup _{\mathbb {R}^N} \left( u_0-v_0\right) +\sup _{r\ge 0}\left( \omega _{v_{0}}\left( r\right) -\lambda r^{2}/2\right) _{+} \\= & {} \sup _{\mathbb {R}^N} \left( u_0-v_0\right) +\theta \left( \omega _{v_{0}},\lambda \right) , \end{aligned}$$

and we conclude letting $\lambda \rightarrow (\Delta ^+ _{T})^{-1}$.

We begin with the observation that, since constants are solutions of (13),

39 $$\begin{aligned} u \;\le \; \Vert u_0\Vert _{\infty ; {\mathbb {R}} ^N} \ \text {and} \ - v \; \le \; \Vert v_0\Vert _{\infty ; {\mathbb {R}} ^N}. \end{aligned}$$

Next we fix $\delta , \alpha >0$ and $0< \lambda <(\Delta ^+ _{T})^{-1}$ and consider the map

$$\begin{aligned} (x,y,t) \rightarrow u(x,t)-v(y,t) -\Phi ^{\lambda }(x,y,t) -\delta \left( |x|^2 + |y|^2)\right) - \alpha t, \end{aligned}$$

which, in view of (39), achieves its maximum at some $(\hat{x}, \hat{y}, \hat{t}) \in {\mathbb {R}} ^N \times { \mathbb {R}} ^N \times [0,T]$ –note that below to keep the notation simple we omit the dependence of $(\hat{x}, \hat{y}, \hat{t})$ on $\lambda ,\delta ,\alpha$.

Let

$$\begin{aligned} M_{\lambda ,\alpha ,\delta }&:=\max _{{\mathbb {R}}^{N}\times {\mathbb {R}^{N} \times [0,T]}}u(x,t)-v(y,t)-\Phi ^{\lambda }(x,y,t)-\delta \left( |x|^2 + |y|^2)\right) - \alpha t \\&=u(\hat{x},\hat{t})-v(\hat{y},\hat{t})-\Phi ^{\lambda }(\hat{x}, \hat{y}, \hat{t}) -\delta \left( |\hat{x}|^2 + |\hat{y}|^2\right) -\alpha \hat{t}. \end{aligned}$$

The lemma below summarizes a number of important properties of $({\hat{x}}, {\hat{y}}, {\hat{t}})$. Since the arguments in the proof are classical in the theory of viscosity solutions, see for example [1, 2], we omit the details.

### Lemma 2.3

Suppose that the assumptions of Theorem 1.1 hold. Then:40


Next we argue that, for any $\lambda$ in a sufficiently small left-neighborhood of $(\Delta ^+ _{T})^{-1}$, we have $d_g(\hat{x},\hat{y})<\Upsilon$, which yields that the eikonal equation for *e* are valid at these points.

In view of the bound on $d_g^{2}(\hat{x},\hat{y})=e_g(\hat{x},\hat{y})$ that follows from part (ii) of Lemma 2.3, it suffices to choose $\lambda$ so that

$$\begin{aligned} 2({1/\lambda +\Delta _{T}^{-}})\left( \Vert u\Vert _{\infty }+\Vert v\Vert _{\infty } \right) < \Upsilon ^{2}. \end{aligned}$$

Taking into account that we also need ${\Delta ^+_{T}<1/\lambda }$, we are led to the condition

$$\begin{aligned} {\Delta ^+_{T}+\Delta _{T}^{-}<\frac{1}{\lambda } +\Delta _{T}^{-}}\le \frac{1}{ 2\left( \Vert u\Vert _{\infty }+\Vert v\Vert _{\infty } \right) }\Upsilon ^{2}; \end{aligned}$$

and finding such $\lambda$ is possible in view of (14).

If $\hat{t}\in (0,T]$, we use the inequalities satisfied by *u* and *v* in the viscosity sense, noting that to simplify the notation we omit the explicit dependence of derivatives of $\Phi$ on $(\hat{x}, \hat{y}, \hat{t})$, and we find, in view of Lemma 2.2 and the Cauchy-Schwarz’s inequality,

$$\begin{aligned} 0&\ge \Phi _t^\lambda + \alpha - (g^{-1}(\hat{x}) (D_{ x}\Phi ^\lambda + 2 \delta \hat{x}), (D_{ x}\Phi ^\lambda + 2 \delta \hat{x})) \dot{\xi }_{\hat{t}} + (g^{-1}(\hat{y}) (D_{ y}\Phi ^\lambda - 2 \delta \hat{y}),\\&\quad (D_{ y}\Phi ^\lambda - 2 \delta \hat{y})) \dot{\zeta }_{\hat{t}}\\&\ge \alpha - {\Vert \dot{\xi } \Vert }_{\infty ;[0,T]} \left( 2 \delta (g^{-1}(\hat{x}) D_{ x} \Phi ^{\lambda }, D_{ x} \Phi ^\lambda )^{1/2} (g^{-1}(\hat{x}) \hat{x}, \hat{x})^{1/2} + \delta ^2 (g^{-1}(\hat{x}) \hat{x}, \hat{x}) \right) \\&\quad \quad - {\Vert \dot{\zeta } \Vert } _{\infty ;[0,T]} \left( 2 \delta (g^{-1}(\hat{y}) D_{ x} \Phi ^{\lambda }, D_{ y} \Phi ^{\lambda })^{1/2} (g^{-1}(\hat{y}) \hat{y}, \hat{y})^{1/2} + \delta ^2 (g^{-1}(\hat{y}) \hat{y}, \hat{y}) \right) . \end{aligned}$$

Using again Lemma 2.3 (i)–(iii), we can now let $\delta$ $\rightarrow$ 0 to obtain $\alpha \le 0$, which is a contradiction.

It follows that, for all $\delta$ small enough, we must have $\hat{t} = 0$ and, hence,

$$\begin{aligned} M_{\lambda ,\alpha ,\delta }\le \left( u_{0}( \hat{x})-v_{0}(\hat{y})-\lambda e(\hat{x},\hat{y})\right) \le \sup _{\mathbb {R}^N}\left( u_{0}-v_{0}\right) +\theta \left( \omega _{u_{0}} \wedge \omega _{v_{0}},\lambda \right) . \end{aligned}$$

Letting first $\delta \rightarrow 0$ and then $\alpha \rightarrow 0$, concludes the proof of (38). $\square$

## The second-order case: the proof of Theorem 1.2

Since the proof of Theorem 1.2 is in many places very similar to that of Theorem 1.1, we omit arguments that follow along straightforward modifications.

In the next lemma we introduce the modified test functions, which here will depend on an additional parameter $\gamma$ corresponding to a time exponential. Since its proof is similar to the one of Lemma 2.2, we omit it.

### Lemma 3.1

Fix $T, \lambda >0, \gamma \ge 0,\,\xi ,\zeta \in C^{1}([ 0,T]; {\mathbb {R}} ^N)$ with $\xi _{0}=\zeta _{0}=0$ and assume that $\lambda \Delta _T^{\gamma ;+} <1.$ Then

$$\begin{aligned} \Phi ^{\lambda ,\gamma }(x,y,t):=\frac{\lambda e^{\gamma t}}{1-\lambda \int _{0}^{t}e^{\gamma s}(\dot{\xi }_{s}-\dot{\zeta }_{s})ds}e_g(x,y) \end{aligned}$$

is a classical solution, in $\left\{ (x,y) \in {\mathbb {R}} ^N\times {\mathbb {R}} ^N: d\left( x,y\right) <\Upsilon \right\} \times [0,T]$, of

$$\begin{aligned} w_t - \gamma w- (g^{-1}(x) D_{x}w, D_{x}w)\dot{\xi } + (g^{-1}(y) D_{y}w, D_{y}w)\dot{\zeta }=0. \end{aligned}$$

Next we specify the range of $\lambda$’s we will use. We set

41 $$\begin{aligned} \bar{\lambda } := (\Delta ^{\gamma , +}_T)^{-1} \ \text {and} \ \underline{\lambda }:= \frac{4K}{\Upsilon - 4K\Delta ^{\gamma ,-}_T}, \end{aligned}$$

and observe that, in view of our assumptions, we have $\bar{\lambda } > \underline{\lambda }.$ We say that $\lambda$ is admissible for fixed $\gamma$ and $\alpha$, if $\lambda \in (\underline{\lambda }, \bar{\lambda })$.

Also note that, if *u*, *v*, $u_{0},v_{0}, \xi$, $\zeta$ and *F* are as in the statement of Theorem 1.2, then

42 $$\begin{aligned} \sup _{\mathbb {R}^N \times [0,T]} (u - v) \le K. \end{aligned}$$

For fixed $\delta >0$ and $\lambda$ admissible we consider the map

$$\begin{aligned} (x,y,t) \rightarrow u(x,t)-v(y,t) -\Phi ^{\lambda ,\gamma }(x,y,t) -\delta \left( |x|^2 + |y|^2)\right) , \end{aligned}$$

which, in view of (39), achieves its maximum at some $(\hat{x}, \hat{y}, \hat{t}) \in {\mathbb {R}} ^N \times { \mathbb {R}} ^N \times [0,T]$ –as before to keep the notation simple we omit the dependence of $(\hat{x}, \hat{y}, \hat{t})$ on $\lambda ,\delta$.

Let

43 $$\begin{aligned} M_{\lambda ,\gamma ,\delta }:= & {} \max _{\left( x,y, t\right) \in {\mathbb {R}}^{N}\times {\mathbb {R}}^{N}\times [0,T]}u(x,t)-v(y,t)-\Phi ^{\lambda ,\gamma }(x,y,t)-\delta (|x|^{2}+|y|^{2})\nonumber \\ \\= & {} u(\hat{x},\hat{t})-v(\hat{y},\hat{t})-\Phi ^{\lambda ,\gamma }( \hat{x},\hat{y},\hat{t})-\delta (|\hat{x}|^{2}+|\hat{y}|^{2}). \nonumber \end{aligned}$$

The following claim is the analogue of Lemma 2.3. As before when writing $\Phi$ and its derivatives we omit their arguments.

### Lemma 3.2

Under the assumptions of Theorem 1.2 and for $\lambda$ admissible we have:

$$\begin{aligned} \left\{ \begin{array}{l} \mathrm{(i)} \ \lim _{\delta \rightarrow 0}\delta (|\hat{x}|^{2}+|\hat{y}|^{2})=0, \\ \mathrm{(ii)} \ e_g(\hat{x},\hat{y})\le 2 K ( \frac{1}{\lambda }+\Delta ^{\gamma ,-}_T ), \\ \mathrm{(iii)} \ |D_{{x}}\Phi ^{\lambda ,\gamma }|^{2}\le \frac{2 \lambda e^{\gamma T}}{1-\lambda \Delta ^{\gamma ;+}_T} K\, \text {and}\\ \mathrm{(iv)} \ \lim _{\delta \rightarrow 0}M_{\lambda ,\gamma ,\delta }=M_{\lambda ,\gamma ,0}. \end{array}\right. \end{aligned}$$

### Proof of Theorem 1.2

If, for some sequence $\delta \rightarrow 0$, $\hat{t}=0$, then

44 $$\begin{aligned} M_{\lambda ,\gamma ,0}=\lim _{\delta \rightarrow 0} M_{\lambda ,\gamma ,\delta } \le u_{0}(\hat{x})-v_{0}(\hat{y})-\Phi ^{\lambda ,\gamma }(\hat{x}, \hat{y},0) \le \Vert \left( u_{0}-v_{0}\right) _{+}\Vert _{\infty }+\theta ( \omega _{u_{0}}\wedge \omega _{v_{0}},\lambda ) . \end{aligned}$$

We now treat the case where $\hat{t}\in (0,T]$ for all $\delta$ small enough.

Since, in view of Lemma 3.2(ii) and the assumptions (recalling that $\lambda$ is admissible), the test-function $\Phi ^{\lambda , \gamma }$ is smooth at $(\hat{x}, \hat{y}, \hat{t} )$, it follows from the theory of viscosity solutions (see, for example, [2]) that

45 $$\begin{aligned} 0\ge & {} {\Phi }^{\lambda , \gamma }_t-F(X+2\delta I, D_{x}{\Phi }^{\lambda , \gamma } +2\delta \hat{x}, u(\hat{x},\hat{t}), \hat{x}, \hat{t})\nonumber \\&-\,(g^{-1}(\hat{x})(D_{{x}}{\Phi }^{\lambda , \gamma } +2\delta \hat{x}), D_{x}{\Phi }^{\lambda , \gamma } +2\delta \hat{x}) \dot{\xi }_{\hat{t}} \nonumber \\&+\,F(Y-2\delta I, -D_{y}{\Phi }^{\lambda , \gamma } -2\delta \hat{y}, v(\hat{y}, \hat{t}), \hat{y}, \hat{t})\nonumber \\&+\, (g^{-1}(\hat{y})(D_{y}{\Phi }^{\lambda , \gamma } +2\delta \hat{y}), D_{y}{\Phi }^{\lambda , \gamma } +2\delta \hat{y})\dot{\zeta }_{\hat{t}}, \end{aligned}$$

where $X, Y \in \mathcal {S}^N$ are such that for a given $\varepsilon >0$,

46 $$\begin{aligned} -\left( \frac{\hat{\alpha }^2}{\varepsilon }+\hat{\alpha }|D^{2}e_g(\hat{x}, \hat{y})|\right) \left( \begin{array}{cc} I\; &{} 0 \\ 0\; &{} I \end{array} \right) \le \;\;\left( \begin{array}{cc} X &{} 0 \\ 0 &{} -Y \end{array} \right) \le \hat{\alpha }D^{2}e_g(\hat{x},\hat{y})+\varepsilon (D^{2}e_g(\hat{x},\hat{y}))^{2} \end{aligned}$$

and

47 $$\begin{aligned} {\hat{\alpha }}:=\frac{\lambda e^{\gamma \hat{t}}}{1-\lambda \int _{0}^{\hat{t}}e^{\gamma s}(\dot{\xi }_{s}-\dot{\zeta }_{s})ds}=\frac{{\Phi }^{\lambda , \gamma }(\hat{x}, \hat{y}, \hat{t})}{e_g( \hat{x},\hat{y})}. \end{aligned}$$

Then, as in the usual proof of the comparison of viscosity solutions, combining (45) and (23), we get that

48 $$\begin{aligned} \rho (u({\hat{x}}, {\hat{t}})-v({\hat{y}}, {\hat{t}}))_+ \le (a)+(b)+(c)+(d), \end{aligned}$$

where

49 $$\begin{aligned} (a):= & {} -F(X, D_{x}\Phi ^{\lambda , \gamma }, u( \hat{x}, \hat{t}), \hat{x}, \hat{t})+F(X+2\delta , D_{x}\Phi ^{\lambda , \gamma }+2\delta \hat{x}, u(\hat{x}, \hat{t}), \hat{x}, \hat{t}), \end{aligned}$$

50 $$\begin{aligned} (b):= & {} F(Y, - D_{y}\Phi ^{\lambda , \gamma }, v( \hat{y}, \hat{t}), \hat{x}, \hat{t})-F(Y+2\delta I, - D_{y}\Phi ^{\lambda , \gamma } -2\delta \hat{y}, v( \hat{y}, \hat{t}), \hat{x}, \hat{t}), \end{aligned}$$

51 $$\begin{aligned} (c):= & {} {\left\{ \begin{array}{ll} \Phi ^{\lambda , \gamma }_t+\gamma \Phi ^{\lambda , \gamma }+(g^{-1}(\hat{x}) (D_{{x}}{ \Phi }^{\lambda , \gamma } +2\delta \hat{x}), D_{{x}}{ \Phi }^{\lambda , \gamma } +2\delta \hat{x})\dot{\xi }_{\hat{t}}\\ - (g^{-1}(\hat{y}) (D_{{y}}{ \Phi }^{\lambda , \gamma }-2\delta \hat{y}), D_{{y}}{ \Phi }^{\lambda , \gamma }-2\delta \hat{y})\dot{\zeta }_{\hat{t}}, \end{array}\right. } \end{aligned}$$

and

52 $$\begin{aligned} (d):=-\gamma \Phi ^{\lambda , \gamma }+F( X, D_{x}\Phi ^{\lambda , \gamma }, u( \hat{x}, \hat{t}), \hat{x}, \hat{t}) -F(Y, - D_{y}\Phi ^{\lambda , \gamma }, v( \hat{y}, \hat{t}), \hat{y}, \hat{t}). \end{aligned}$$

Since (17) and (46) imply that *X* and *Y* stay bounded, in view of (21), we get $\limsup _{\delta \rightarrow 0}((a)+(b))=0$.

Moreover, the quadratic form of the equation satisfied by $\Phi ^{\lambda , \gamma } =\hat{\alpha }e_g$ gives

$$\begin{aligned} (c) \le C \delta |D_{\hat{x}}\Phi ^{\lambda , \gamma } | ( |\hat{x}| + |\hat{y}|) \left( {\Vert \dot{\xi } \Vert }_{\infty ;[0,T]} + {\Vert \dot{\zeta } \Vert } _{\infty ;[0,T]}\right) , \end{aligned}$$

and using Lemma 3.2 (i),(iii) we find $\lim _{\delta \rightarrow 0}(c)=0$.

For the last term, note that Lemma 3.2 (ii) and (22) yield, always at the point $(\hat{x}, \hat{y}, \hat{t})$,

$$\begin{aligned} (d)= & {} -\gamma \hat{\alpha }{e_g}+ F( X, \hat{\alpha }D_{x} e_g, u( \hat{x}, \hat{t}), \hat{x}, \hat{t}) -F(Y, -\hat{\alpha }D_{y} e_g, v(\hat{y}, \hat{t}), \hat{y}, \hat{t})\\\le & {} -\frac{\gamma \hat{\alpha }}{2}d^{2}( \hat{x},\hat{y}) +\omega _{F, K}\left( d\left( \hat{x},\hat{y}\right) +\hat{\alpha }d^{2}\left( \hat{x},\hat{y}\right) + \varepsilon \right) \\\le & {} -\frac{\gamma \hat{\alpha }}{2}d^{2}\left( \hat{x},\hat{y}\right) +\omega _{F, K}(\hat{\alpha }d^{2}( \hat{x},\hat{y})) + \omega _{F,K} (d( \hat{x},\hat{y}) + \omega _{F, K}\left( \varepsilon \right) \\\le & {} \tilde{\theta }\left( \omega _{F,K},\gamma \right) + \omega _{F, K} (2 (K (\frac{1}{\lambda } + \Delta ^{\gamma ;-})^{1/2}) + \omega _{F, K}\left( \varepsilon \right) . \end{aligned}$$

Combining the last four estimates and (44) and letting $\varepsilon \rightarrow 0$ we find that, for all $\lambda \in (\underline{\lambda },\bar{\lambda })$

$$\begin{aligned} u(x,t)-v(x, t)\le & {} M_{\lambda ,\gamma ,0}= \lim _{\delta \rightarrow 0}M_{\lambda ,\gamma ,\delta } \\\le & {} \left\| \left( u_{0}-v_{0}\right) _{+}\right\| _{\infty }+ \theta \left( \omega _{u_{0}}\wedge \omega _{v_{0}},\lambda \right) \\&+ \frac{1}{\rho }\tilde{\theta }\left( \omega _{F,K},\gamma \right) + \frac{1}{\rho } \omega _{F,K} \left( 2 (K \left( \frac{1}{\lambda } + \Delta ^{\gamma ;-}_T\right) ^{1/2}\right) . \end{aligned}$$

Letting $\lambda \rightarrow \bar{\lambda }$ and using the continuity of $\theta$ in the last argument, we finally obtain that, for all $\gamma \in \Gamma$,

$$\begin{aligned} u-v\le & {} \left\| \left( u_{0}-v_{0}\right) _{+}\right\| _{\infty }+\theta \left( \omega _{u_{0}}\wedge \omega _{v_{0}}, (\Delta ^{\gamma ;+}_T)^{-1} \right) +\frac{1}{\rho }\tilde{\theta } \left( \omega _{F,K}; \gamma \right) \\&+ \frac{1}{\rho } \omega _{F,K}\left( 2 (K (\frac{1}{\lambda } + \Delta ^{\gamma ;-}_T)^{1/2}\right) . \end{aligned}$$

$$\square $$

## The properties of the geodesic energy and the assumptions of Theorem 1.2

In this section we prove that $C^2_b$-bounds on *g* and $g^{-1}$ imply (17) and verify that the *F* in (30), if (32) and (33) hold, satisfies the assumptions of Theorem 1.2.

We begin with the former.

### Proposition 4.1

Assume (7). Then there exists $\Upsilon >0$ such that, in the set $\left\{ (x,y): d_{g}(x,y)<\Upsilon \right\}$, $e_g$ is twice continuously differentiable and (17) is satisfied with bounds depending only on appropriate norms of $g,g^{-1}$.

### Proof

We begin by recalling some basic facts concerning geodesics and distances.

For each fixed point *x*, there is a unique geodesic with starting velocity $v=\dot{\gamma }\left( 0\right)$ given by $\gamma _{t}= X_{t}$, where $\left( X,P\right) _{t\ge 0}$ is the solution to the characteristic equations

53 $$\begin{aligned} {\left\{ \begin{array}{ll} \dot{X}_{s}= 2g^{-1}(X_s) P_s \;\;\;\;\;X_{0}=x, \\ \dot{P}_{s}=- (Dg^{-1}(X_s)P_s,P_s)\;\;\;\;\;P_{0}=p= \tfrac{1}{2} g(x)v. \end{array}\right. } \end{aligned}$$

Equivalently $(\gamma )_{t \ge 0}$ satisfies the second order system of ode

54 $$\begin{aligned} \ddot{\gamma }_{t}+\Gamma _{ij}^{k}\left( \gamma _{t}\right) \dot{\gamma } _{t}^{i}\dot{\gamma }_{t}^{j}=0 \ \ \ \gamma _{0}=x, \ \dot{\gamma }_{0}=v \end{aligned}$$

with

55 $$\begin{aligned} \Gamma _{ij}^{k}:=g^{k\ell }\left( \partial _{i}g_{\ell j}+\partial _{j}g_{\ell i}-\partial _{\ell }g_{ij}\right) \text {.} \end{aligned}$$

It is easy to see that (53) has a global solution $\left( X,P\right) _{t\ge 0}$, since, in view of (6) and (7) as well as the invariance of the flow, we have, for $t\ge 0$,

56 $$\begin{aligned} | P_t | \approx (g^{-1}(X_t)P_t, P_t) = (g^{-1}(X_0)P_0, P_0) \approx |p|^2. \end{aligned}$$

As a consequence, the projected end-point map $E_{x}\left( p\right) :=X_{1}( x, p)$ is well-defined for any *p*.

We note that the energy along a geodesic $\gamma$ emerging from $\gamma _0 = x$ has a simple expression in terms of $p=P_0$ or $v= \dot{\gamma }_{0}$. Indeed, the invariance of the Hamilonian $H(x,p) = (g^{-1}(x)p,p)$ under the flow yields

57 $$\begin{aligned} |\dot{\gamma }_{0}|^2_g := ( g\left( x\right) v,v) = 4 \, H(x,p) = 4 \int _{0}^{1} H(X_t,P_t) dt= \int _{0}^{1}(g\left( \gamma _{t}\right) \dot{\gamma } _{t},\dot{\gamma }_{t}) dt. \end{aligned}$$

It is a basic fact that distance minimizing curves (geodesics) are also energy minimizing.

Indeed, given *x*, *y*, (53) and equivalently (54), are the first-order optimality necessary conditions for these minimization problems. Hence, in view of (57),

$$\begin{aligned} e_{g}\left( x,y\right)&= \inf \left\{ \frac{1}{4}\left| \dot{\gamma } _{0}\right| _{g}^{2}:\gamma \text { satisfies (54) } \text { with} \ \gamma _{0}=x \text { and } \gamma _{1}=y\right\} \\&= \inf \big \{ H(X_0,P_0) : (X,P) \text { satisfies (53) } \text { with} \ X_{0}=x \text { and } X_{1}=y\big \} . \end{aligned}$$

A standard compactness argument implies the existence of at least one geodesic connecting two given points *x*, *y*. In general, however, more than one geodesic from *x* to *y* may exist, each determined by its initial velocity $\dot{\gamma }_{0}=v$ or equivalently $P_0 = p = \frac{1}{2} g(x) v$, upon departure from *x*.

It turns out that, for *y* close to *x*, there exists exactly one geodesic. Indeed, if $g^{-1} \in C^2$, it is clear from (53) that $E_x = (p \mapsto X_1(x,p))$ has $C^1$-dependence in *p*. Since $D_{p} E_{x} (p)$ is non-degenerate in a neighborhood of $p=0$, it follows from the inverse function theorem that, for *y* close enough to *x*, one can solve $y=E_x(p)$ uniquely for $p=E_{x}^{-1}(y)$ with $C^1$-dependence in *y*. Hence

58 $$\begin{aligned} e_g (x,y) = H (x, p) = H(x, E_{x}^{-1}(y)). \end{aligned}$$

The gradient $D_{x}e_g\left( x,y\right)$ points in the direction of maximal increase of $x\mapsto e\left( x,y\right)$. Since $E_{x}^{-1}\left( y\right) =p$ is precisely the co-velocity of *X* at $X_{0}=x$ and *X* is the geodesic from *x* to $X_{1}=y$, it follows that

59 $$\begin{aligned} D_{x}e_g\left( x,y\right) =-E_{x}^{-1}y=-p. \end{aligned}$$

This easily implies that, for points *x*, *y* close enough, the energy has continuous second derivatives. Indeed, existence of continuous mixed derivatives $D^2_{xy} e_g$ follows immediately from the $C^1$-regularity of $E_{x}^{-1}=E_{x}^{-1}(y)$. Concerning $D^2_{xx} e_g$ (and by symmetry $D^2_{yy}e_g$) we set $p=p_0$ above and note that by exchanging the roles of *x*, *y*, we have $D_{y}e_g\left( x,y\right) =-E_{y}^{-1}\left( x\right) =P_{1}\left( x,p_{0}\right) =:p_{1}$ and so, from (53),

60 $$\begin{aligned} D_{y}e_g\left( x,y\right) +D_{x}e_g\left( x,y\right) = p_{1}-p_{0}=-\int _{0}^{1}H_{x}\left( X_{s},P_{s}\right) ds. \end{aligned}$$

The existence and continuity of $D^2_{xx} e_g$ is then clear, since the right-hand side above has $C^1$-dependence in *x* as is immediate from (53) and $g^{-1} \in C^2$.

All the above considerations have been so far local. It is hence necessary to address the (global) question of regularity in a strip around the diagonal $\{ x = y \}$. For this we need to control $\alpha _1:=D_p E_x (p) = D_p X_1 (x,p)$. We do this by considering the tangent flow

$$\begin{aligned} (\alpha _t, \beta _t) := (D_p X_t(x,p),D_p P_t(x,p)), \end{aligned}$$

which solves the matrix-valued linear ode

61 $$\begin{aligned} {\left\{ \begin{array}{ll} \dot{\alpha }_{s}=2Dg^{-1}(X_s) P_s \alpha _{s}+ 2g^{-1}(X_s)\beta _{s},\;\;\;\;\;\alpha _{0}=0, \\ \dot{\beta }_{s}=- (D^2g^{-1}(X_s)P_s, P_s) \alpha _{s}- 2 \, Dg^{-1}(X_s) P_s \beta _{s},\;\;\;\;\;\beta _{0}=I . \end{array}\right. } \end{aligned}$$

We now argue that, uniformly in *x*,

$$\begin{aligned} D_p E_x (p) = \alpha _1 \approx 2 \, g^{-1} (x). \end{aligned}$$

It follows that $D_p E_x$ is non-degenerate, again uniformly in *x*. Indeed if $\alpha _0 = 0$, whenever *p* is small, $X_\cdot \approx x, \beta _\cdot \approx I$ and we have

$$\begin{aligned} \left( \begin{array}{c} \dot{\alpha } \\ \dot{\beta } \end{array} \right) =\left( \begin{array}{ll} \text {(small)} &{} 2 g^{-1} (X_\cdot ) \\ \text {(small)} &{} \, \, \text {(small)} \end{array} \right) \left( \begin{array}{c} \alpha \\ \beta \end{array} \right) \, . \end{aligned}$$

Next we prove the above claim. First, it follows from (56) that, if *p* is small, then $$P_t(x,p)$$ stays small, over, for example, a unit time interval [0, 1]. Moreover, since $$\dot{X} = 2 \, g^{-1} (X) P$$, the boundedness of $$g^{-1}$$ yields that the path $$X_\cdot (x, p)$$ also stays close to $$X_0 = x$$, again uniformly on [0, 1]. Furthermore, the $$C^1$$- and $$C^2$$-bounds on $$g^{-1}$$ yield that the matrices $$Dg^{-1}(X_s) P_s$$ and $$ (D^2g^{-1}(X_s)P_s, P_s)$$ are small along $$(X_{s},P_{s})$$, while $$2g^{-1}(X_s)$$ is plainly bounded. This implies that $$\alpha _\cdot $$ and $$\beta _\cdot $$ will stay bounded. Then $$\dot{\beta }_\cdot $$ will be small, and, hence, $$\beta _\cdot $$ will be close to $$\beta _0 = I$$, uniformly on [0, 1]. In turn, $$\dot{\alpha }_s$$ is the sum of a small term plus $$ 2 \ g^{-1}(X_s)\beta _{s} \approx 2 g^{-1} (x)$$. In other words,

$$\begin{aligned} \dot{\alpha }_s = 2 g^{-1}(x) + \Theta _s(x,p), \end{aligned}$$

where

$$\begin{aligned} \lim _{\delta \rightarrow 0} \sup _{|p|\le \delta } \sup _{s \in [0,1]} \sup _{x \in \mathbb {R}^N} \left| \Theta _s(x,p) \right| = 0. \end{aligned}$$

Since $D_p E_x (p) = \alpha _1 = \int _0^1 \dot{\alpha }_s ds$, it follows that, there exists some $\delta >0$, which can be taken proportional to $M^{-4}$ where $M=1+ \Vert g \Vert _{C^{2}} +\Vert g^{-1}\Vert _{C^{2}}$, such that

$$\begin{aligned} | D_p E_x (p) - 2 g^{-1} (x) | \le \Vert g\Vert _\infty ^{-1} \text{ for } \text{ all } x,p \in \mathbb {R}^N, |p| \le \delta . \end{aligned}$$

Note that the choice of the constant $\Vert g\Vert _\infty ^{-1}>0$ on the right-hand side guarantees that $D_p E_x (p)$ remains non-degenerate, uniformly in *x*.

It follows by the inverse function theorem that $p \mapsto E_{x}(p)$ is a diffeomorphism from $B_\delta$ onto a neighbourhood of *x*. We claim that this neighbourhood contains a ball of radius $\Upsilon >0$, which may be taken independent of *x*.

For this we observe that, with $p = E_x^{-1}(y)$, (58) yields

$$\begin{aligned} d_g(x,y) = \sqrt{e_g (x, y)} = \sqrt{ H (x, p) } = \sqrt{ (g^{-1}(x) p, p) } . \end{aligned}$$

Hence it suffices to choose $\Upsilon >0$ small enough so that $(g^{-1}(x) p,p) \le \Upsilon ^2$ implies $\left| p \right| \le \delta$, an obvious choice being $\Upsilon = \delta /c$, where $c^2$ is the ellipticity constant of *g*.

At last, we note that (59), in conjunction with the just obtained quantitative bounds, implies that $D^{2}e_{g}$ is bounded on $\left\{ (x,y)\in \mathbb {R}^N\times \mathbb {R}^N: d_{g}(x,y)<\Upsilon \right\}$. Indeed, with $p=E_x^{-1}(y)$, we have $-D^2_{xy} e_g(x,y) = D_y E_x^{-1}(y) = (D_p E_x(p))^{-1} \approx \frac{1}{2} g(x)$ which readily leads to bounds of the second mixed derivatives, uniformly over *x*, *y* of distance at most $\Upsilon$. Similar uniform bounds for $D^2_{xx} e_g$ (and then $D^2_{yy} e_g$) are obtained by differentiating (60) with respect to *x* and estimating the resulting right-hand side. $\square$

The comparison proofs in the viscosity theory typically employ the quadratic penalty function $\phi \left( x,y\right) =\frac{1}{2}\left| x-y\right| ^{2}$ and make use of (trivial) identities such as

$$\begin{aligned} D_{x}\phi +D_{y}\phi =(x-y)+(y-x)=0 \end{aligned}$$

and

$$\begin{aligned} \left( \begin{array}{cc} D^2_{xx}\phi &{} D^2_{xy}\phi \\ D^2_{yx}\phi &{} D^2_{yy}\phi \end{array} \right) = \left( \begin{array}{cc} I &{} -I \\ -I &{} I \end{array} \right) , \,\, \, \,\,\left( p,\,q\right) \left( \begin{array}{cc} D^2_{xx}\phi &{} D^2_{xy}\phi \\ D^2_{yx}\phi &{} D^2_{yy}\phi \end{array} \right) \left( \begin{array}{c} p \\ q \end{array} \right) \le \left| p-q\right| ^{2}\text {.} \end{aligned}$$

To see what one can expect in more general settings, consider first the case of *g* obtained from the Euclidean metric, written in different coordinates, say $x=\Psi ^{-1}\left( \tilde{x}\right)$, in which case, we have

$$\begin{aligned} e_g\left( x,y\right) =\left| \Psi \left( x\right) -\Psi \left( y\right) \right| ^{2}. \end{aligned}$$

If $\Psi$ is bounded in $C^{2}$, it is immediate that

$$\begin{aligned} D_{x}e+D_{y}e= & {} (\Psi \left( x\right) -\Psi \left( y\right) )D\Psi \left( x\right) +(\Psi \left( y\right) -\Psi \left( x\right) )D\Psi \left( y\right) \\= & {} (\Psi \left( x\right) -\Psi \left( y\right) ) \left( D\Psi \left( y\right) -D\Psi \left( y\right) \right) , \end{aligned}$$

and, hence,

62 $$\begin{aligned} \left| D_{x}e+D_{y}e\right| \lesssim \left| x-y\right| ^{2} \end{aligned}$$

and, similarly,

$$\begin{aligned} \left( p,\,q\right) \left( \begin{array}{cc} D^2_{xx}e &{} D^2_{xy}e \\ D^2_{yx}e &{} D^2_{yy}e \end{array} \right) \left( \begin{array}{c} p \\ q \end{array} \right) \lesssim \left| p-q\right| ^{2}+\left| x-y\right| ^{2}. \end{aligned}$$

Unfortunately no such arguments work in the case of general Riemannian metric, since, in general, there is no change of variables of the form $\left| \Psi \left( x\right) -\Psi \left( y\right) \right|$ that reduces $d_{g}\left( x,y\right)$ to a Euclidean distance.

The next two propositions provide estimates that can be used in the comparison proofs in place of the exact identities above.

### Proposition 4.2

Assume (7). Then there exists $\Upsilon >0$ such that whenever $d_g(x,y) < \Upsilon$,

63 $$\begin{aligned} \left| D_{x}e_g+D_{y}e_g\right| \le L \left| x-y\right| ^{2} \end{aligned}$$

with a constant *L* that depends only on the $C^{1}$-bounds of $g,g^{-1}$.

### Proof

As pointed out in the proof of Proposition , for all $(x,y): d_{g}(x,y)<\Upsilon$,

$$\begin{aligned} D_{y}e_g\left( x,y\right) +D_{x}e_g\left( x,y\right) = -\int _{0}^{1} ( Dg^{-1}(X_{s})P_{s},P_{s} ) ds. \end{aligned}$$

Using that $g^{-1}\in C^{1}$ and the fact that *g* is uniformly comparable to the Euclidean metric we get

$$\begin{aligned} \left| D_{y}e\left( x,y\right) +D_{x}e\left( x,y\right) \right|\le & {} \Vert g^{-1}\Vert _{C^{1}}\int _{0}^{1}\left| P_{s}\right| ^{2}ds \\\le & {} \Vert g^{-1} \Vert _{C^{1}} \Vert g\Vert _{\infty }\int _{0}^{1}( g^{-1}\left( X_{s}\right) P_{s},P_{s}) ds \end{aligned}$$

and, hence, thanks to invariance of the Hamiltonian under the flow and (58),

$$\begin{aligned} \left| D_{y}e\left( x,y\right) +D_{x}e\left( x,y\right) \right| \lesssim \int _{0}^{1}H(X_{s},P_{s})ds=H\left( X_{0},P_{0}\right) = e_g \left( x,y\right) . \end{aligned}$$

Using again that *g* is uniformly comparable to the Euclidean metric the proof is finished. $\square$

The following claim applies in particular under condition (7), which, in view of Propostion 4.1, implies $C^2$-regularity of the energy near the diagonal. The proof is based on an argument in a forthcoming paper by the last two authors [11].

### Proposition 4.3

Assume there exists $\Upsilon >0$ such that, in the set $\left\{ (x,y): d_{g}(x,y)<\Upsilon \right\}$, $e_g$ is twice continuously differentiable. Then, whenever $d_g(x,y) < \Upsilon$,

$$\begin{aligned} \left( \begin{array}{cc} D_{xx}^{2}e_{g} &{} D_{xy}^{2}e_{g} \\ D_{yx}^{2}e_{g} &{} D_{yy}^{2}e_{g} \end{array} \right) \le L\left( \begin{array}{cc} I_{N} &{} -I_{N} \\ -I_{N} &{} I_{N} \end{array} \right) +L\left| x-y\right| ^{2}I_{2N} \end{aligned}$$

with a constant *L* that only depends on the $C^{2}$-bounds of $g,g^{-1}$.

### Proof

In view of the assumed $C^{2}$-regularity of the energy, we find that, as $\varepsilon \rightarrow 0$,

$$\begin{aligned} {\left\{ \begin{array}{ll} \varepsilon ^{2}\left( \left( p,q\right) \left( \begin{array}{cc} D_{xx}^{2}e_{g} &{} D_{xy}^{2}e_{g} \\ D_{yx}^{2}e_{g} &{} D_{yy}^{2}e_{g} \end{array} \right) \left( \begin{array}{c} p \\ q \end{array} \right) \right) \\ =e_{g}\left( x+\varepsilon p,y+\varepsilon q\right) +e_{g}\left( x-\varepsilon p,y-\varepsilon w\right) -2e_{g}\left( x,y\right) \end{array}\right. } \end{aligned}$$

We estimate the second-order difference on the right-hand side, keeping $v=\varepsilon p,w=\varepsilon q$ fixed.

Let $(\gamma _t)_{t\in [0,1]}$ be a geodesic connecting *x*, *y*, parametrized at constant speed (in the metric *g*) so that

64 $$\begin{aligned} \left| \dot{\gamma }_{t}\right| \le c\left( g\left( \gamma _{t}\right) \dot{\gamma }_{t},\dot{\gamma }_{t}\right) ^{1/2}= 2cd_{g}\left( x,y\right) \le C\left| x-y\right| , \end{aligned}$$

and, for $\Delta :=w-v$, define the paths

$$\begin{aligned} \gamma _{t}^{\pm } : =\gamma _{t}\pm \left( v+t\Delta \right) \end{aligned}$$

which connect $x\pm v$ to $y\pm w$.

Then

$$\begin{aligned}&e\left( x+v,y+w\right) +e\left( x-v,y-w\right) -2e\left( x,y\right) \\&\quad \le \int _{0}^{1}\frac{1}{4}\left( g\left( \gamma _{t}^{+}\right) \dot{ \gamma }_{t}^{+},\dot{\gamma }_{t}^{+}\right) dt+\int _{0}^{1}\frac{1}{4}\left( g\left( \gamma _{t}^{-}\right) \dot{\gamma }_{t}^{-},\dot{\gamma } _{t}^{-}\right) dt-2\int _{0}^{1}\frac{1}{4}\left( g\left( \gamma _{t}\right) \dot{\gamma }_{t},\dot{\gamma }_{t}\right) dt \\&\quad =\frac{1}{4}\int _{0}^{1}\left[ \left( g\left( \gamma _{t}^{+}\right) \left( \dot{\gamma }_{t}+\Delta \right) ,\dot{\gamma }_{t}+\Delta \right) +\left( g\left( \gamma _{t}^{-}\right) \left( \dot{\gamma }_{t}-\Delta \right) ,\dot{\gamma }_{t}-\Delta \right) dt-2\left( g\left( \gamma _{t}\right) \dot{\gamma }_{t},\dot{\gamma }_{t}\right) \right] dt \end{aligned}$$

Using the $C^{2}$-regularity of *g*, writing $\delta =v+t\Delta$, and noting that that $\left| \delta \right| \le \left| v\right| +\left| w\right|$, we find

$$\begin{aligned} g\left( \gamma _{t}^{\pm }\right) =g\left( \gamma _{t}\right) \pm \left( Dg\left( \gamma _{t}\right) ,\delta \right) +\frac{1}{2}\left( D^{2}g\left( \gamma _{t}\right) \delta ,\delta \right) +o\left( \left| v\right| ^{2}+\left| w\right| ^{2}\right) . \end{aligned}$$

Collecting terms (in $g,Dg,D^{2}g$) then leads to

$$\begin{aligned} e\left( x+v,y+w\right) +e\left( x-v,y-w\right) -2e\left( x,y\right) \le \frac{1}{4}\int _{0}^{1}\left[ \left( i\right) +\left( ii\right) +\left( iii\right) +\left( E\right) \right] dt \end{aligned}$$

where

$$\begin{aligned} {\left\{ \begin{array}{ll} \left( i\right) :=2\left( g\left( \gamma _{t}\right) \Delta ,\Delta \right) \\ \left( ii\right) :=4\left( \left( Dg\left( \gamma _{t}\right) ,\delta \right) \dot{\gamma }_{t},\Delta \right) \\ \left( iii\right) :=\left( \left( D^{2}g\left( \gamma _{t}\right) \delta ,\delta \right) \dot{\gamma }_{t},\dot{\gamma }_{t}\right) +\left( \left( D^{2}g\left( \gamma _{t}\right) \delta ,\delta \right) \Delta ,\Delta \right) \end{array}\right. } \end{aligned}$$

It is immediate that,

$$\begin{aligned} {\left\{ \begin{array}{ll} \left| \left( i\right) \right| \le 2\left\| g\right\| _{\infty }\left| w-v\right| ^{2}\\ \left| \left( ii\right) \right| \le 4C\left\| Dg\right\| _{\infty }\left( \left| v\right| +\left| w\right| \right) \left| x-y\right| \left| w-v\right| \\ \left| iii\right| \le \left\| D^{2}g\right\| _{\infty }\left( \left| v\right| ^{2}+\left| w\right| ^{2}\right) \left( C^{2}\left| x-y\right| ^{2}+\left| w-v\right| ^{2}\right) . \end{array}\right. } \end{aligned}$$

Moreover, expanding *g*, as $v,w\rightarrow 0$, we find

$$\begin{aligned} \left( E\right) =(\left| \dot{\gamma }_{t}\right| ^{2}+\left| \Delta \right| ^{2}) o\left( \left| v\right| ^{2}+\left| w\right| ^{2}\right) =(C^{2}\left| x-y\right| ^{2}+\left| w-v\right| ^{2}) o\left( \left| v\right| ^{2}+\left| w\right| ^{2}\right) . \end{aligned}$$

With $v=\varepsilon p,w=\varepsilon q$ all terms above are of order $O\left( \varepsilon ^{2}\right)$, with the exception of the second term contributing to $\left( iii\right)$ and the error term $\left( E\right)$ which are actually $o\left( \varepsilon ^{2}\right)$, and hence negligible as $\varepsilon \rightarrow 0$.

Indeed

$$\begin{aligned}&e( x+\varepsilon p,y+\varepsilon q) +e( x-\varepsilon p,y-\varepsilon w) -2e( x,y) \\&\quad \le \frac{\varepsilon ^{2}}{4}[ 2\Vert g\Vert _{\infty }\vert p-q \vert ^{2}+4C\Vert Dg\Vert _{\infty }( \vert p\vert +\vert q\vert ) \vert x-y\vert \vert p-q\vert \\&\quad \quad +\Vert D^{2}g\Vert _{\infty }( \vert p\vert ^{2}+\vert q\vert ^{2}) ( C^{2}\vert x-y\vert ^{2}+O( \varepsilon ^{2})) +(\vert x-y\vert ^{2}+O( \varepsilon ^{2}) )o( 1)] . \end{aligned}$$

Using again the Cauchy–Schwarz inequality to handle the middle term in the estimate, we find, for some $K>0$ that only depends on *g*,

$$\begin{aligned}&\left( p,q\right) \left( \begin{array}{cc} D_{xx}^{2}e_{g} &{} D_{xy}^{2}e_{g} \\ D_{yx}^{2}e_{g} &{} D_{yy}^{2}e_{g} \end{array} \right) \left( \begin{array}{c} p \\ q \end{array} \right) \\&\quad \le \Big [ \frac{1}{2}\left\| g\right\| _{\infty }\left| p-q\right| ^{2}+C\left\| Dg\right\| _{\infty }\left( \left| p\right| +\left| q\right| \right) \left| x-y\right| \left| p-q\right| \\&\quad \quad +\frac{C^{2}}{4}\left\| D^{2}g\right\| _{\infty }( \left| p\right| ^{2}+\left| q\right| ^{2}) \left| x-y\right| ^{2}\Big ] \\&\quad \le K\left| p-q\right| ^{2}+K\left| x-y\right| ^{2}\left( \left| p\right| ^{2}+\left| q^{2}\right| \right) . \end{aligned}$$

$$\square $$

We conclude by checking that the assumptions of Theorem 1.2 are satisfied for *F* of Hamilton-Jacobi-Isaacs type. Note that condition (7) is valid, as demanded by that theorem.

### Proposition 4.4

Let *F* be given by (30), satisfying (32) and (33). Then *F* satisfies (18)–(22).

### Proof

Since it is clear that all assumptions are stable under taking sup and inf, we will only treat the quasilinear case

$$\begin{aligned} F(M,p,r,x) = {\text {Tr}}(a(p,x)M) + b(p,x) - c(x) r, \end{aligned}$$

and we concentrate on (22), since the others are obvious.

With $t,x,y,r,\alpha ,X,Y$ taken as in the statement of the assumption, we have

$$\begin{aligned}&F(X, \alpha D_{x}d_g^{2}(x,y), r,x,t)-F(Y,-\alpha D_{y}d_g^{2}(x,y),r,x,t)\\&\quad = b\left( p,x \right) - b\left( q,y \right) + \mathrm {Tr}\left( \sigma \sigma ^T(p,x) X - \sigma \sigma ^T(q,y) Y\right) + (c(x)-c(y))r, \end{aligned}$$

where for simplicity we write $p: =\alpha D_{x}d_g^{2}(x,y)$ and $q:=-\alpha D_{y}d_g^{2}(x,y)$.

Noting that Proposition 4.2 yields

$$\begin{aligned} |p-q| \le \alpha K |x-y|^2, \end{aligned}$$

and using the eikonal equation for $e_g = d_g^2$, for some $C>0$ we find

$$\begin{aligned} \alpha C^{-1} |x-y| \le |p| + |q| \le \alpha C |x-y|. \end{aligned}$$

The assumptions on *b* also give

$$\begin{aligned} b\left( p,x \right) - b\left( q,y\right)\le & {} \omega \big ( \left( 1 + |p| + |q| \right) |x-y| +|p-q|\big ) \\\le & {} \omega \left( \left( 1+C \alpha |x-y|\right) |x-y| + K \alpha |x-y|^2 \right) . \end{aligned}$$

For the second order term, we use Proposition 4.3 and get

$$\begin{aligned} \mathrm {Tr}\left( \sigma \sigma ^T(p,x) X - \sigma \sigma ^T(q,y) Y\right)= & {} (\sigma (p,x) , \, \sigma (q,y)) \begin{pmatrix} X &{} 0 \\ 0 &{} -Y \end{pmatrix} \begin{pmatrix} \sigma (p,x) \\ \sigma (q,y) \end{pmatrix}\\\le & {} \alpha \left( \sigma (p,x), \, \sigma (q,y)\right) \left( \begin{array}{cc} D^2_{xx}e &{} D^2_{xy}e \\ D^2_{yx}e &{} D^2_{yy}e \end{array} \right) \left( \begin{array}{c} \sigma (p,x) \\ \sigma (q,y) \end{array} \right) \\&+ \;\; \varepsilon \left\| \left( \begin{array}{cc} D^2_{xx}e &{} D^2_{xy}e \\ D^2_{yx}e &{} D^2_{yy}e \end{array} \right) \left( \begin{array}{c} \sigma (p,x) \\ \sigma (q,y) \end{array} \right) \right\| ^2 \\\le & {} K \alpha ( \left| \sigma (p,x) - \sigma (q,y)\right| ^{2}+\left| x-y\right| ^{2}) + K \varepsilon \\\le & {} K^{\prime }\alpha \left( |x-y|^2 + \frac{|p-q|^2}{(|p|+|q|)^2} \right) + K^{\prime } \varepsilon \\\le & {} K^{\prime \prime } \left( \alpha |x-y|^2 + \varepsilon \right) . \end{aligned}$$

$$\square $$
